# Supplementary material for: Robust negative longitudinal magnetoresistance and spin–orbit torque in sputtered Pt3Sn and Pt3SnxFe1-x topological semimetal
Source: Nat Commun. 2023 Jul 12;14:4151. doi: 10.1038/s41467-023-39408-2 (PMC10338453; doi:10.1038/s41467-023-39408-2)
Supplement: Supplementary file 1 — Supplementary Information [file 41467_2023_39408_MOESM1_ESM.pdf]

Supplementary Information for

Robust negative longitudinal magnetoresistance and spin-orbit torque in sputtered Pt_3Sn topological semimetal

Delin Zhang,[1*†] Wei Jiang,[1*] Hwanhui Yun,[2] Onri Jay Benally,[1] Thomas Peterson,[3] Zach Cresswell,[1] Guichuan Yu,[4] Javier Garcia Barriocanal,[4] Przemyslaw Swatek[1], K. Andre Mkhoyan,[2] Tony Low,[1†] Jian-Ping Wang[1,2,3†]

[1]Department of Electrical and Computer Engineering, University of Minnesota, Minneapolis, MN, 55455, USA; [2]Department of Chemical Engineering and Materials Science, University of Minnesota, Minneapolis, MN, 55455, USA ; [3]School of Physics and Astronomy, University of Minnesota, Minneapolis, MN, 55455, USA; [4]Characterization Facility, University of Minnesota, Minneapolis, MN, 55455, USA.

*These authors contributed equally to this work.

†Corresponding authors. jpwang@umn.edu (J.P.W.), tlow@umn.edu (T.L.) and dlzhang@umn.edu (D.L.Z.)

This supplementary information includes:

Supplementary note 1: Structural characterization of Pt_3Sn, $Pt_3Sn_xFe_{1-x}$ and Mo-seeded Pt_3Sn

Supplementary note 2: Composition and magnetic properties of $Pt_3Sn_xFe_{1-x}$

Supplementary note 3: Three-resistor model for negative longitudinal MR

Supplementary note 4: Phase transition from WTI to WSM

Supplementary note 5: Measurement of Spin torque efficiency θ_{SH}

Figs: S1-S12

Supplementary Note 1. Structural characterization of Pt_3Sn, $Pt_3Sn_xFe_{1-x}$ and Mo-seeded Pt_3Sn

To investigate the microstructure and chemical composition of the Pt_3Sn thin films, we carried out cross-sectional STEM analysis of the Pt_3Sn and $Pt_3Sn_xFe_{1-x}$samples. Fig. S2(a) shows a Pt_3Sn thin film and capping layers deposited on a (001) MgO single crystalline substrate. The sample is a homogeneous film with sharp interfaces between the constituent layers. The Pt_3Sn thin film does not follow the texture of the (001) MgO substrate. The films mainly grow along the (111) crystalline orientation and contain small grains with the (001) orientation. HAADF-STEM image of a (001) oriented grain embedded in the majority of (111) oriented grains is shown on the right. Fe-doped Pt_3Sn ($Pt_3Sn_xFe_{1-x}$) sample also has the same crystalline structure as Pt_3Sn (Fig. S2(b)). The atomic-resolution HAADF-STEM images of (111) oriented grains projected from the $[1\bar{1}0]$ and $[11\bar{2}]$ directions are presented on the right. Comparison of the EDX spectra from the Pt_3Sn and $Pt_3Sn_xFe_{1-x}$ thin films reveals that the relative amount of Sn is lower in $Pt_3Sn_xFe_{1-x}$, which indicates that Fe substituting Sn atoms. Fig. S2(c) presents the results of the STEM-EDX analysis of the Mo-seeded Pt_3Sn/CoFeB sample, where the Mo seed layer follows the (001) texture of a MgO substrate and induces the (001) texture of Pt_3Sn. Here, Sn diffusion at the Mo/Pt_3Sn interface is also observed, which is attributed to sample heating.

The electric-transport properties of the Pt-seeded Pt_3Sn and Mo-seeded Pt_3Sn samples were also tested. The resistivity (ρ_{xx}) is measured and calculated to be ~145 μΩ.cm and ~192 μΩ.cm for Pt-seeded Pt_3Sn and Mo-seeded Pt_3Sn at room temperature, respectively, as plotted in Fig. S3(a). With decreasing the testing temperature, ρ_{xx} varies in a metallic manner, reaching at 1.9 K a residual value ρ_0 of about ~52 μΩ.cm and ~108 μΩ.cm for Pt-seeded Pt_3Sn and Mo-seeded

Pt_3Sn, respectively, which arises due to charge scattering on lattice defects. The residual resistivity ratio RRR = ρ(300 K)/ρ(0) ~2-3 signals high quality of the studied thin film materials.

Supplementary Note 2. Composition and magnetic property of $Pt_3Sn_xFe_{1-x}$

To investigate the magnetic properties of $Pt_3Sn_xFe_{1-x}$ samples, we characterized the composition and temperature-dependent magnetic-hysteresis (*M-H*) loops of $Pt_3Sn_xFe_{1-x}$ by RBS and PPMS with VSM mode. The composition of $Pt_3Sn_xFe_{1-x}$ is calculated to be Pt:Sn:Fe=75.2:21.1:3.8. The magnetic property was measured as a function of temperature. Fig. S4 shows the raw data of the *M-H* loops with the magnetic field along in-plane and out-of-plane. We find that the Fe dopant induces magnetism in the $Pt_{75.2}Sn_{21.1}Fe_{3.8}$ thin film. Because the magnetic field is not enough to saturate the sample, we did not subtract the background contribution for $Pt_{75.2}Sn_{21.1}Fe_{3.8}$ thin film. From Figs. S4(a) and S4(b), we can observe that the magnetic property will vanish when the temperature reaches 25 K. Below 25 K, it shows ferromagnetic properties.

Supplementary Note 3. Three-resistor model and NLMR

To understand the NLMR behavior of the experimentally grown Pt_3Sn films, we applied a three-resistor model to fit with the measured MR results. The resistance of the trivial metallic states [see Fig. S6(a)] can be well described by the Drude's form $R_c(H_{ext}) = R_{c,0}[1+\alpha(H_{ext}\cdot sin(\theta))^2]$, while that of the topological semimetal states [see Fig. S6(b)] is assumed to be $R_{SM}(H_{ext}) = R_{SM,0}[1+\beta(H_{ext}\cdot cos(\theta)\cdot cos(\varphi))^2]$. The difference between the two cases is mainly due to the sign of the parameter in front of H_{ext}, i.e., α and β in units of Oe^{-2}. A positive/negative parameter will yield a negative/positive MR, which could be further enhanced with the increase of the absolute value of such parameters, as shown in Figs. S6(a) and S6(b), respectively. We note that, for the time reversal semimetals with fixed Weyl pair

distribution, we can roughly use 0 and 90^0 to represent E∥B and E⊥B, respectively. However, for the TRS-broken WSM or polycrystalline systems with various crystal orientations, $R_{SM}(H_{ext})$ becomes almost angle-independent as Weyl pair distribution is correlated with the external magnetic field, so we chose a constant for this scenario. Because of the coexistence of trivial bulk states, Dirac fermions, and topological surface states in the experimentally grown Pt_3Sn thin films through sputtering, multiple MR could be affecting the signals simultaneously. More specifically, those semimetal states around the Fermi level will contribute to the negative MR signals, while the trivial metallic state will contribute positive MR signals.

Furthermore, we tried to fit the experimental data with this proposed three-resistor model. We note that the weak antilocalization effect is not considered in our model, which may not fully reproduce all the details of the MR results, especially in the very low field region. However, the model should still be valid to capture the essential physics for the NLMR behavior. To fit with the experiments, we assumed that R_c^s and/or R_c^p are the same and determine the resistance $R_{c,0}$ and $R_{SM,0}$ at zero field, and finally tune both α and β to compare with the experiments. One important feature of the three-resistor model is that R_{SM} usually plays a more dominant role for low H_{ext} region, leading to the formation of NLMR. While R_c^s or R_c^p become more important for high H_{ext} region, resulting in eventually a positive MR when H_{ext} is higher than a certain threshold. For different systems, different fitting parameters are obtained. For samples without magnetic doping, there is a clear angle dependence. For pure Pt_3Sn, we get $R_{SM,0}$=0.062 mΩ, $R_{c,0}$ = 0.070 mΩ, α = -1.82×10^{-11} Oe^{-2} and β = 6.35×10^{-12} Oe^{-2}; For Mo/Pt_3Sn, we get $R_{SM,0}$ =0.062 mΩ, $R_{c,0}$ =0.0697 mΩ, α = -0.72×10^{-12} Oe^{-2} and β = 1.85×10^{-11} Oe^{-2}; For Pt/Pt_3Sn, we get $R_{SM,0}$ =0.062 mΩ, $R_{c,0}$ =0.0697 mΩ, α = -5.72×10^{-12} Oe^{-2} and β = 9.55×10^{-12} Oe^{-2}.

With the change of θ between H_{ext} and I_c, the MR changes from negative to positive, which agrees very well with the experimental measurements, as can be seen in Figs. 3(b), S8(a) and S8(c). Using the same set of parameters, when φ is changed, the MR remains negative, as the positive contribution is eliminated. The fitting agrees with the experiments very well, as shown in in Figs. 3(c), S8(b) and S8(d). However, due to the existence of multiple pair of Weyl nodes, experimental negative MR remains non-zero even when φ = 90 degree, while the theoretical model that use an ideal model with one single pair of Weyl nodes show zero MR instead. For $Pt_3Sn_xFe_{1-x}$, as $R_{SM}(H_{ext})$ is weakly dependent on the angle between H_{ext} and I_c and shows a linear behavior, we used a linear model $MR \cong \alpha + \beta H_{ext}$ to fit with experiments. We get α = (0.018-0.030) and β = (3.41-3.93)$\times 10^{-6}$ Oe^{-1} for θ, and α = (0.017-0.019) and β = (3.01-3.31)$\times$ 10^{-6} Oe^{-1} for φ, as plotted in Figs. 3(d) and 3(e). Both MR results with different θ and φ show a decrease of intensity with the increase of angle between H_{ext} and I_c. Here, the larger NLMR is possibly related to the dominating contribution from TRS broken Weyl fermions, compared to competition between positive and negative MR in the time reversal semimetals.

It is known that the suppression of the spin fluctuation in weak metals due to H_{ext} could reduce the electrical resistivity and thus lead to NMR, which is proportional to H_{ext}^2 in the paramagnetic phase and to H_{ext} in the ferromagnetic phase within the weak field region. However, such suppression of spin fluctuation induced NMR is independent of the relative angle between the electric field and H_{ext}, which is clearly different from our experimental observations that only show NMR when the electric field is parallel to H_{ext}. Weak localization could also lead to NMR that could be attributed to local impurities, but it is usually observed only in low H_{ext} range. This is different from our experimental observation for the Pt_3Sn system which has NMR signal in a wide H_{ext} range. In addition, considering the conserved time-reversal symmetry and the high

crystal isotropy, weak localization cannot explain the clear NLMR under parallel H_{ext} either. Such significant angle dependence can be attributed to the chiral anomaly, which also agrees with our theoretical calculations and modeling results.

Supplementary Note 4. Phase transition from WTI to WSM

Pristine Pt_3Sn shows clear topological features near the Fermi level, as confirmed through edge state calculations shown in Fig. S9. However, there is significant bulk band contribution with a type-II Dirac state around the Fermi level, making Pt_3Sn an imperfect topological insulator, which is termed as a weak topological insulator (WTI) or topological semimetal. Such a coexistence of bulk, Dirac, and topological surface state at the Fermi level was usually considered less interesting for studying topological properties, as the bulk state may prevent clear examination of topological state for the measurement of quantum conductance. However, we find the bulk trivial band and Dirac fermion may provide a critical foundation to trigger the topological transition to various WSM phases and therefore surprisingly bring in intriguing chiral anomaly transport behaviors.

Considering the experimental sputtering Pt_3Sn thin films may have various structural distortions, such as point defect, lattice strain, and different interfaces between domain boundaries, it is important to study the influence on the electronic properties of the Pt_3Sn thin films. Therefore, we carried out two calculations with structural distortion, i.e., one with two Sn atoms shifting a small distance towards x direction that breaks the inversion symmetry [see Fig. S10(a)] and the other one with in-plane strain that breaks the structural isotropy [see Fig. S10(c)]. The band structure with/without SOC for the first case are shown in Fig. S10(b) with blue and red lines, respectively. One can clearly see that the Dirac node is shifted away from the R point as in the pristine Pt_3Sn after the structural distortion while SOC is not considered. A similar

effect can be seen near the Γ point. When the SOC is considered, the Dirac node become gapped as expected. Interestingly, the bulk bands are further split and form various nontrivial band crossings near the Fermi level due to the structural distortion, as can be seen in the zoomed-in band structure [see right panel of Fig. S10(b)], indicating the formation of the WSM phase. Similar features can also be seen near the Γ point. We further analyzed the band structure with in-plane strain, as shown in Fig. S10(d). The Dirac node is also shifted away from the R point due to symmetry breaking, however, the band remains degenerate because of the coexistence of inversion symmetry and time-reversal symmetry, forming a Dirac semimetal instead of Weyl semimetal. Such Dirac semimetal systems can also result in NLMR behavior, similar to WSMs.

We further considered the situation with the breaking of TRS. The calculation is performed using the virtual crystal approximation method, where the corner-site Sn atoms are mixed with Fe atoms (5%), as suggested by experimental measurements [see Fig. 4(d)]. Figure 4(e) shows the corresponding band structure. For the case without SOC, the band structure shows clearly spin splitting due to the magnetic doping that breaks the time-reversal symmetry. However, the overall shape of the band structure is almost identical to that of Pt_3Sn, as the high crystal symmetry remains unchanged during calculations. When the SOC effect is considered, one can clearly see the formation of various pairs of Weyl nodes near the R [see Fig. 4(f)] and Γ points, confirming the formation of the TRS-broken WSM phase. Therefore, we have proven the topological transition between topological Dirac semimetal and WSM through either inversion symmetry or time-reversal symmetry breaking for Pt_3Sn and demonstrate the robustness of topological semimetal states against structural perturbation. Note that such transition is much easier for the WTI because of the existence of bulk states at the Fermi level. A small perturbation will be enough to achieve such transformation.

It is important to note that the position of the Weyl nodes is closely related to the way of symmetry breaking. For example, when applying translation action to Pt atoms along x direction, the Dirac node at Γ node shifts towards z direction and forming pairs of Weyl nodes around the new Dirac node after SOC is considered, as shown in Fig. S10(b). Therefore, for the structural distortion induced topological semimetal phase, their Weyl nodes (or degenerated Weyl nodes as in Dirac nodes) distribution is fixed and correlated with the specific way of the structural symmetry breaking, leading to the angle-dependent negative MR behavior. Differently, for the time-reversal symmetry breaking WSMs, their Weyl nodes are bound into with the magnetic orientation, i.e., changes with external magnetic field direction, resulting in almost angle-independent negative MR behavior.

In conventional theory of metals and semiconductors, MR should increase quadratically in weak magnetic fields and then saturate in strong fields, which normally is driven by the Lorentz force and has a positive sign. With the discovery of the chiral anomaly of Dirac fermions, novel negative longitudinal MR (NLMR) behavior is theoretically predicted and experimentally observed in Dirac/Weyl semimetal systems, as also observed in our Pt_3Sn system. Such NLMR usually scales with H^2, which can be linked to the conserved time-reversal symmetry ($\mathcal{T}$) of the Dirac Hamiltonian and its Berry curvature. This could be understood by looking at the expanded magnetoconductivity tensor $\sigma_{33}[H] \cong \sigma_{33}^{(0)} + \sigma_{33}^{(1)}[H] + \sigma_{33}^{(2)}[H^2] + \cdots$, where $\sigma_{33}^{(1)\mathcal{T}} = -\sigma_{33}^{(1)}$ while $\sigma_{33}^{(0)\mathcal{T}} = \sigma_{33}^{(0)}$, and $\sigma_{33}^{(2)\mathcal{T}} = \sigma_{33}^{(2)}$ for time reversal invariant systems, leading to the forbidden linear term, i.e., $\sigma_{33}^{(1)} = 0$, and an overall quadratic LNMR behavior. By breaking the time-reversal symmetry, $\sigma_{33}^{(1)}$ is not restricted to be zero, thus resulting in the linear NLMR. This phenomenon has been numerically calculated in $\mathcal{T}$-breaking Weyl semimetal systems (Refs. 31,32,33).

5. Measurement of Spin torque efficiency θ_{SH}

ST-FMR is a self-calibrated approach to characterize θ_{SH} modeled through the Landau-Lifshitz-Gilbert (LLG) equation with the ST term. When the ferromagnetic layer with an in-plane anisotropy is used to characterize θ_{SH}, the anti-damping torque (ADT, τ_{AD}) originating from the spin Hall effect (SHE) lies in-plane and gives rise to a peak that is symmetric with respect to the resonance field (H_0). The field-like torque (FLD, τ_{FL}) and the Oersted field induced torque (OT, τ_{Oe}) are perpendicular to the film and give rise to an anti-symmetric peak. The symmetric and anti-symmetric parts will satisfy the following equation (52,53):

$$V = V_{Sym}\frac{\Delta H^2}{\Delta H^2 + \left(H - H_0\right)^2} + V_{Asym}\frac{\Delta H\left(H - H_0\right)}{\Delta H^2 + \left(H - H_0\right)^2} \qquad (1)$$

Where H is the external field, H_0 is the resonance field, ΔH is the linewidth of the Lorentzian functions, V_{Sym} denotes the strength of symmetric component, and V_{Asym} the asymmetric component.

Noting that both Oersted field and FLT contribute to the antisymmetric component, we used a thickness dependent method to separate these two contributions. FLT is usually determined by the spin Hall layer, regardless of the thickness of the ferromagnetic layer. However, the Oersted field contribution will change if we vary the thickness of the ferromagnetic layer, which changes the current distribution in the device. We can thus derive the equation which separates the FLT (τ_{FL}) and Oersted field (τ_{Oe}) contribution:

$$\frac{\tau_{FL} + \tau_{Oe}}{\tau_{AD}} = \frac{V_{Asym}}{V_{Sym}}\sqrt{1 + \frac{4\pi M_{eff}}{H_0}} \qquad (2)$$

Where τ_{AD}, V_{Sym}, V_{Asym}, and H_0 are anti-damping torque, amplitude of symmetric, asymmetric components, and resonance field.

In our ST-FMR measurement, we prepared Pt sample as a reference and measured its θ_{SH} for the comparison with the Pt_3Sn samples at the same experimental conditions. Through the ST-FMR measurement, we obtained $\theta_{SH} \sim 0.1$ for Pt sample, as shown in Fig. S11.

The SOT efficiency is affected by many factors, including the band topology, bulk and surface contributions, thermal effects, defect contributions, and so on. With a suitable amount of magnetic doping, the Dirac semimetal can be effectively tuned into Weyl semimetal, similar to the topological transition between quantum anomalous Hall insulator and spin Hall insulator in Bi_2Se_3 systems. However, possibly due to the very weak doping effect, the variation of the band topology around the Fermi level is rather small, which leads to only a small variation of the SOT efficiency. This is also consistent with our theoretical calculations, which show small variations of the band structure before and after the magnetic doping. We note that the crystal quality also plays an important role in determining the SOT efficiency, as suggested by the different SOT efficiency in different Pt_3Sn samples grown on distinct substrates.

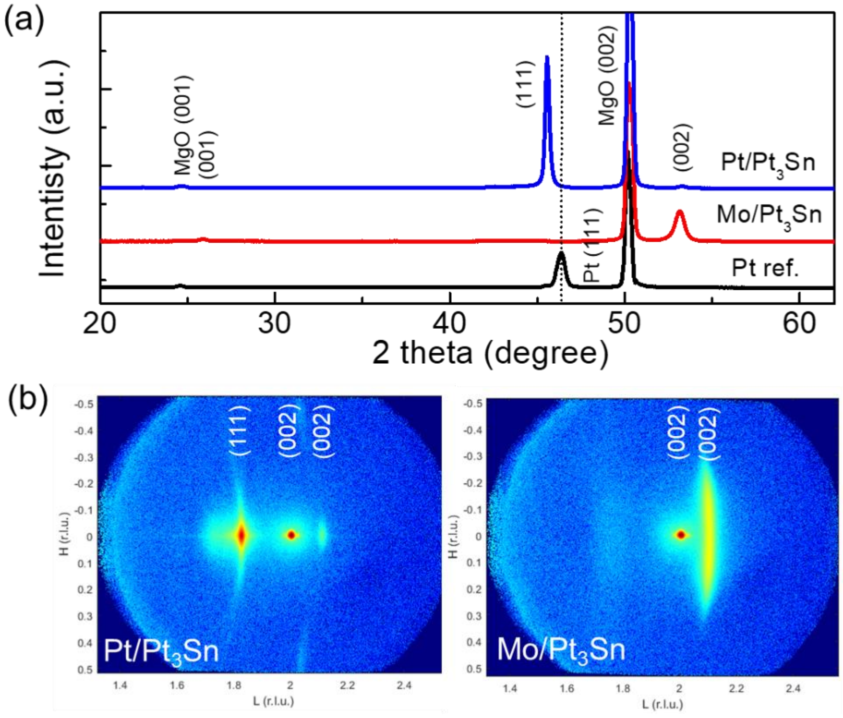

Fig. S1. (a) The out-of-plane (θ-2θ scans) XRD patterns and **(b)** reciprocal space mapping (RSM) of the Pt-seeded Pt_3Sn and Mo-seeded Pt_3Sn thin films. Compared to the (111) direction growth of Pt-seeded Pt_3Sn, Mo-seeded Pt_3Sn shows the (002) textured growth.

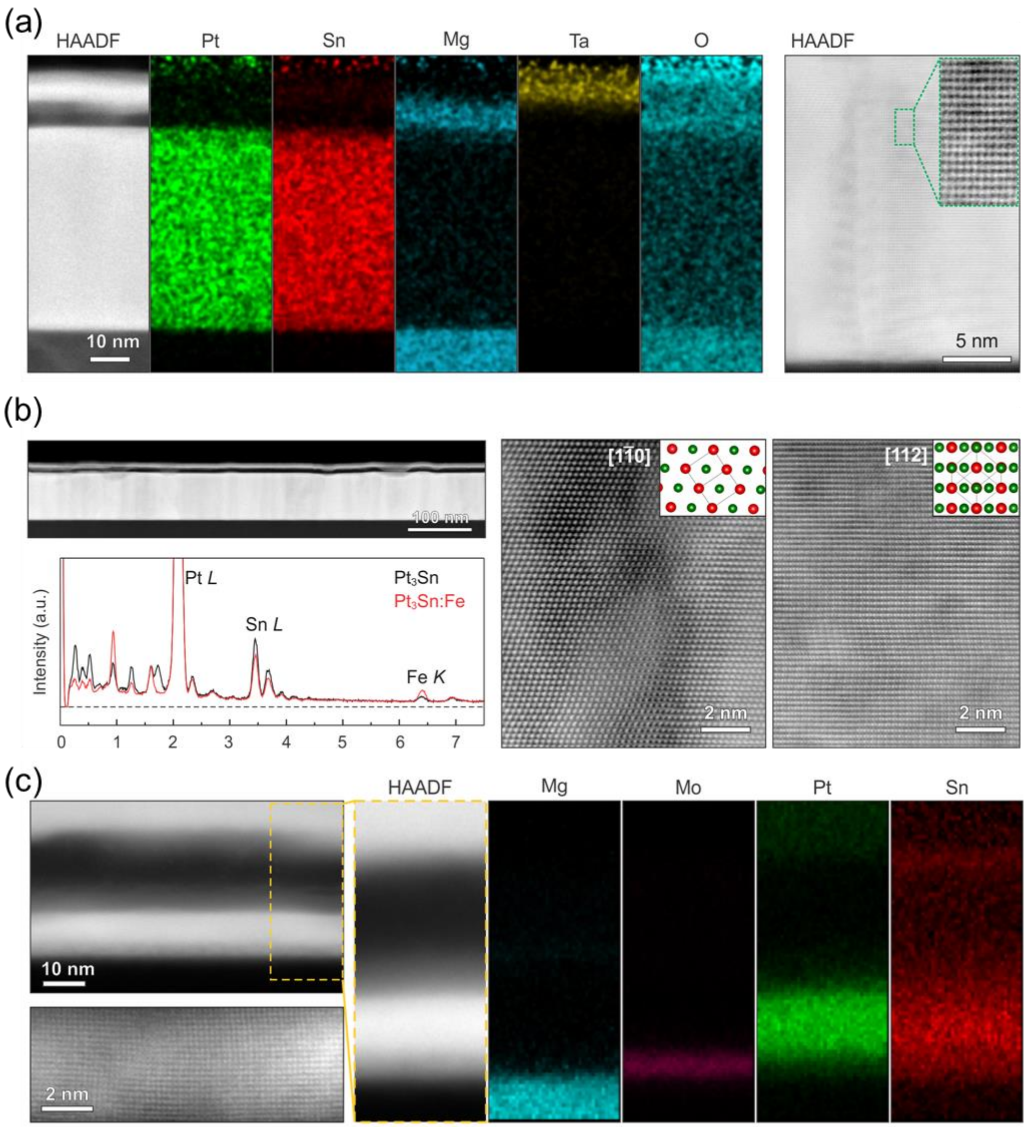

Fig. S2. (a) HAADF-STEM image and complimentary EDX elemental maps of the MgO(sub.)/Pt_3Sn/MgO/Ta system. HAADF-STEM image of a (001) oriented grain embedded in (111) oriented grains is shown on the right. **(b)** STEM-EDX analysis of MgO(sub.)/$Pt_3Sn_xFe_{1-x}$/MgO/Ta system including low-magnification HAADF-STEM image (top), EDX spectra obtained from the $Pt_3Sn_xFe_{1-x}$ and Pt_3Sn thin films (bottom), and atomic-resolution HAADF-STEM images of (111) oriented grains viewed from two different zone axes, [110] and [112]. **(c)** HAADF-STEM images and complimentary EDX elemental maps of the MgO(sub.)/Mo-seed Pt_3Sn/CoFeB system.

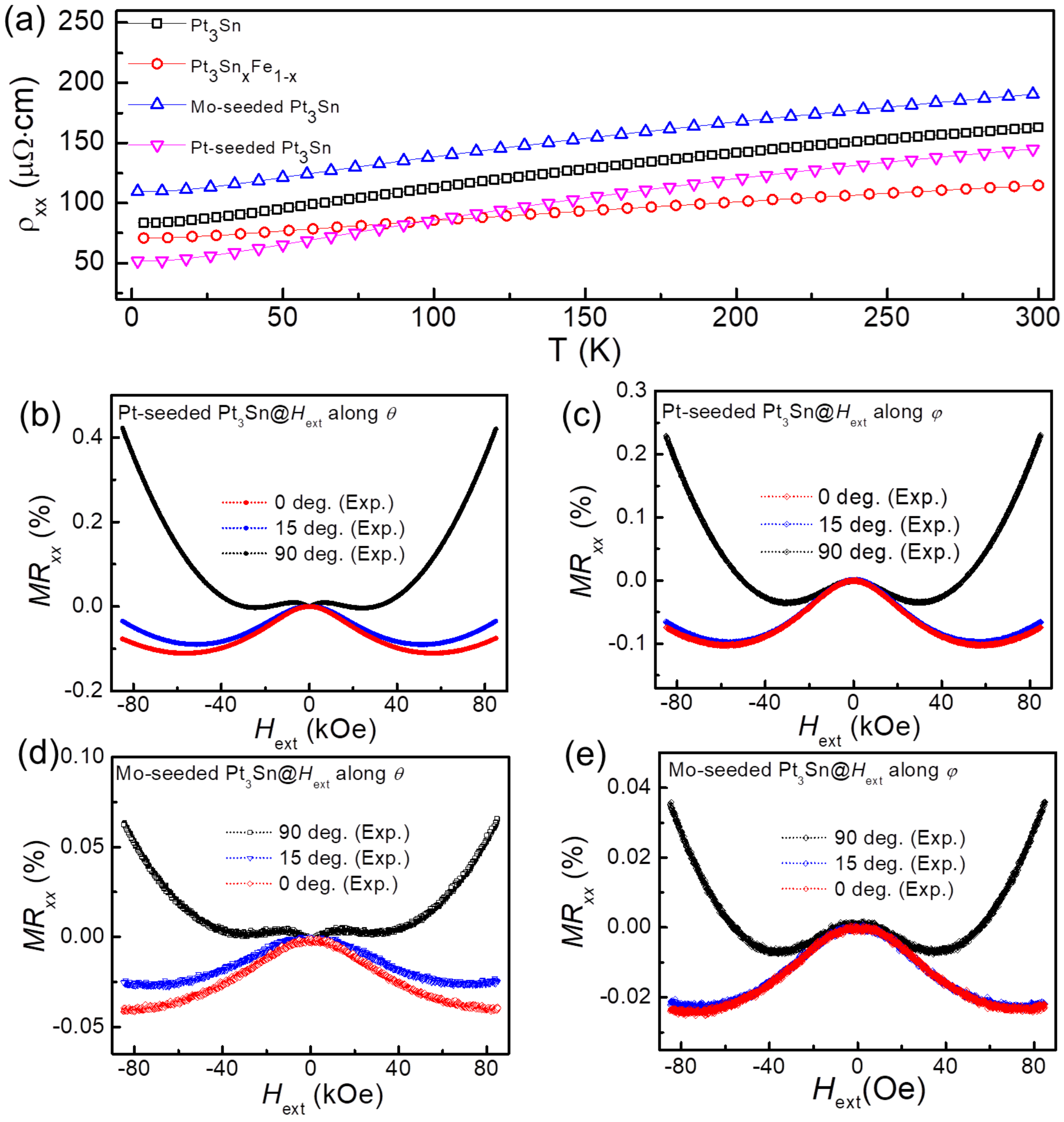

Fig. S3. (a) The resistivity (ρ_{xx}) vs. temperature (T) curves of Pt_3Sn, $Pt_3Sn_xFe_{1-x}$, Pt-seeded Pt_3Sn and Mo-seeded Pt_3Sn. MR_{xx} vs. H_{ext} curves were measured at 1.9 K with H_{ext} along out-of-plane rotation (θ = 0°, 15°, 90°) and in-plane rotation (φ = 0°, 15°, 90°): **(b), (c)** Pt-seeded Pt_3Sn; **(d), (e)** Mo-seeded Pt_3Sn, respectively.

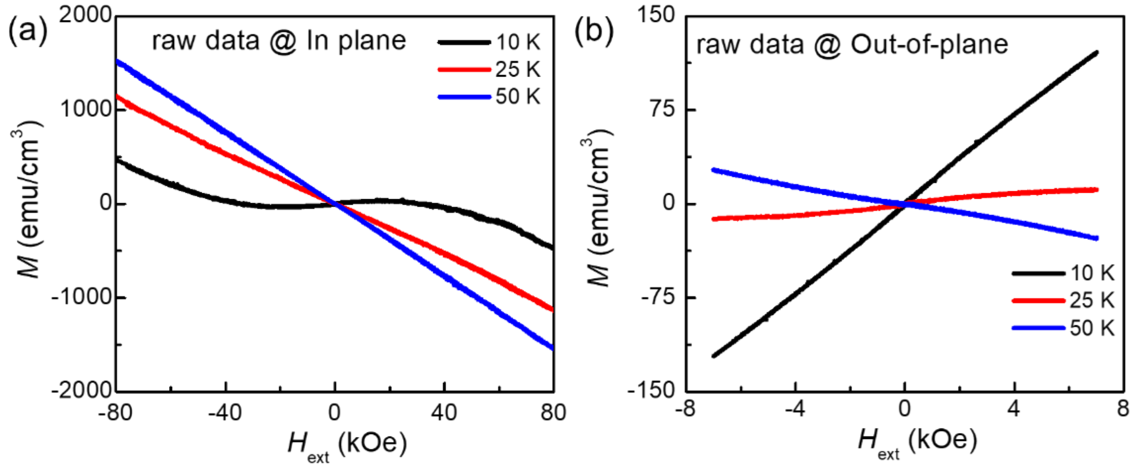

Fig. S4. (**a**) (**b**) The raw *M*-*H* loops of the 60-nm thick $Pt_3Sn_xFe_{1-x}$ thin film measured with in-plane and out-of-plane external magnetic field, respectively.

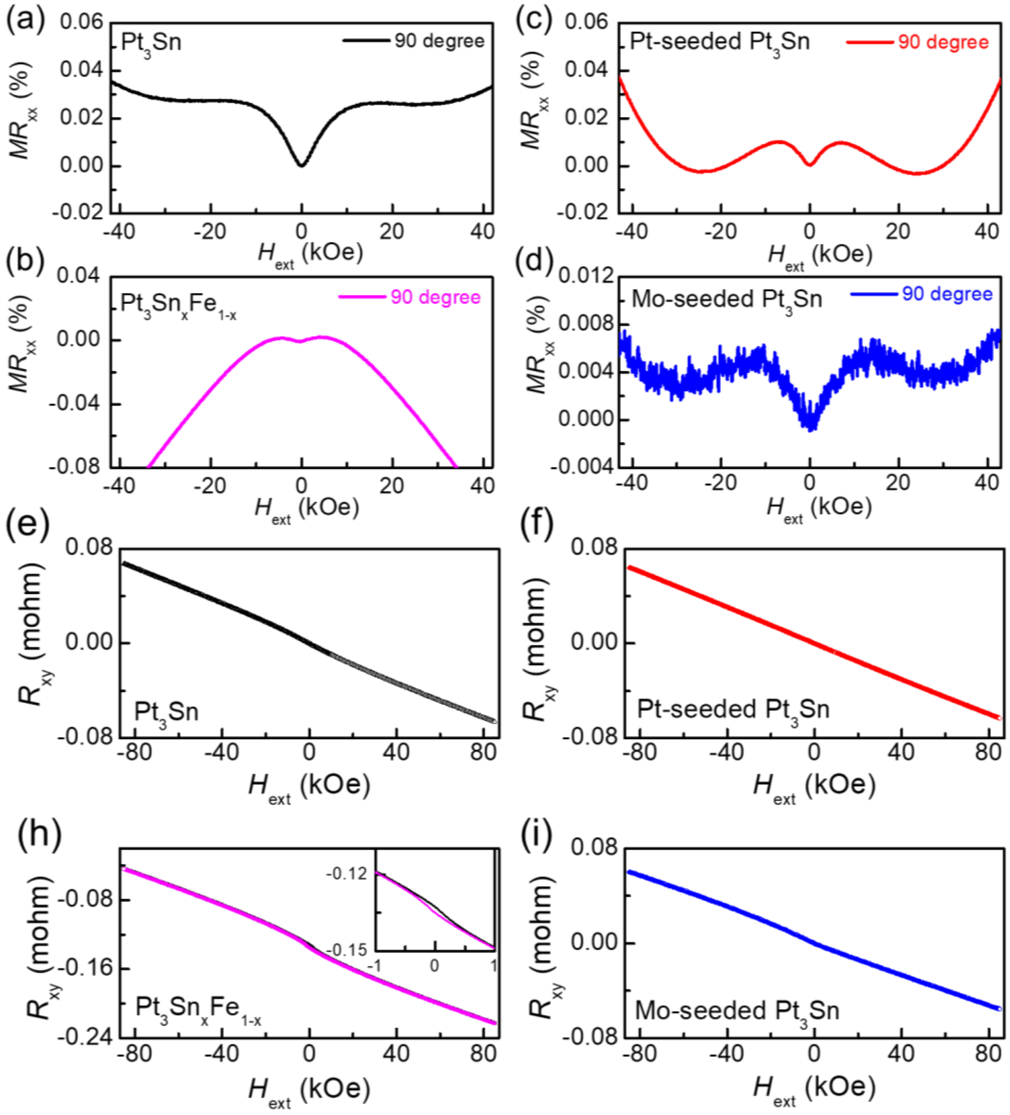

Fig. S5. The zoomed-in MR_{xx} vs. H_{ext} curves measured at 1.9 K with θ = 90 degree. **(a)** Pt_3Sn, **(b)** $Pt_3Sn_xFe_{1-x}$, **(c)** Pt-seeded Pt_3Sn, and **(d)** Mo-seeded Pt_3Sn. From these figures, we can clearly observe the weak anti-localization behavior, suggesting topological properties. The Hall resistance (R_{xy}) vs. external magnetic field (H_{ext}) curves measured at 1.9 K with θ = 90 degree. **(e)** Pt_3Sn, **(f)** $Pt_3Sn_xFe_{1-x}$, **(h)** Pt-seeded Pt_3Sn, and **(i)** Mo-seeded Pt_3Sn.

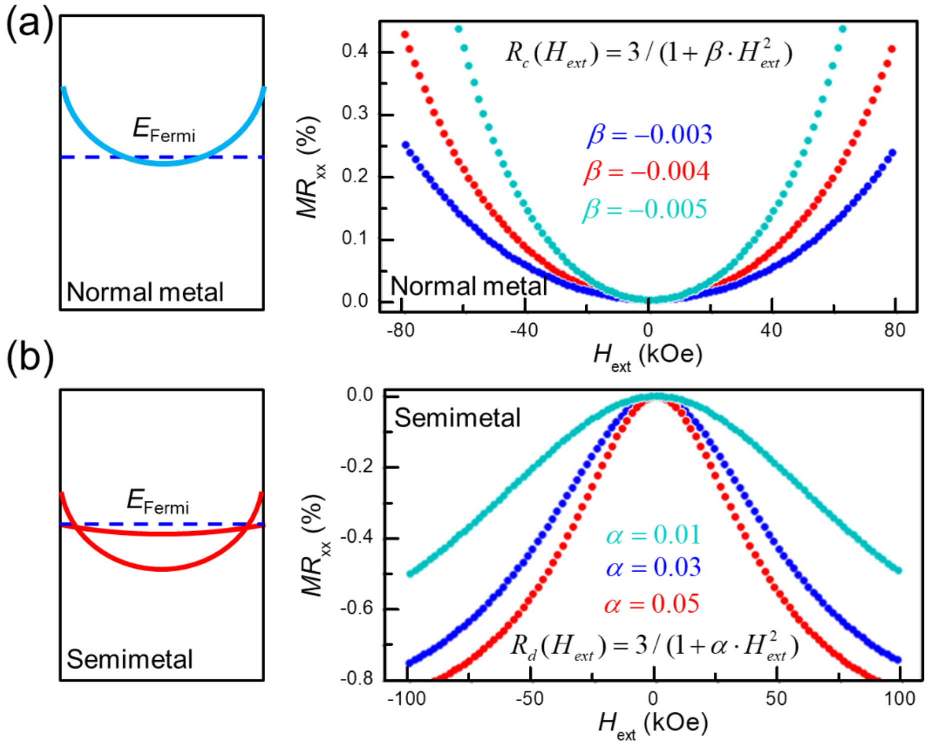

Fig. S6. Simulation of MR for normal metal and Semimetal through three-resistor model. **(a)** Schematic band structure (left panel) and MR results (right panel) for the normal metal using Drude model with different parameters. **(b)** Schematic band structure (left panel) and MR results (right panel) for semimetals using Drude model with different parameters having opposite sign.

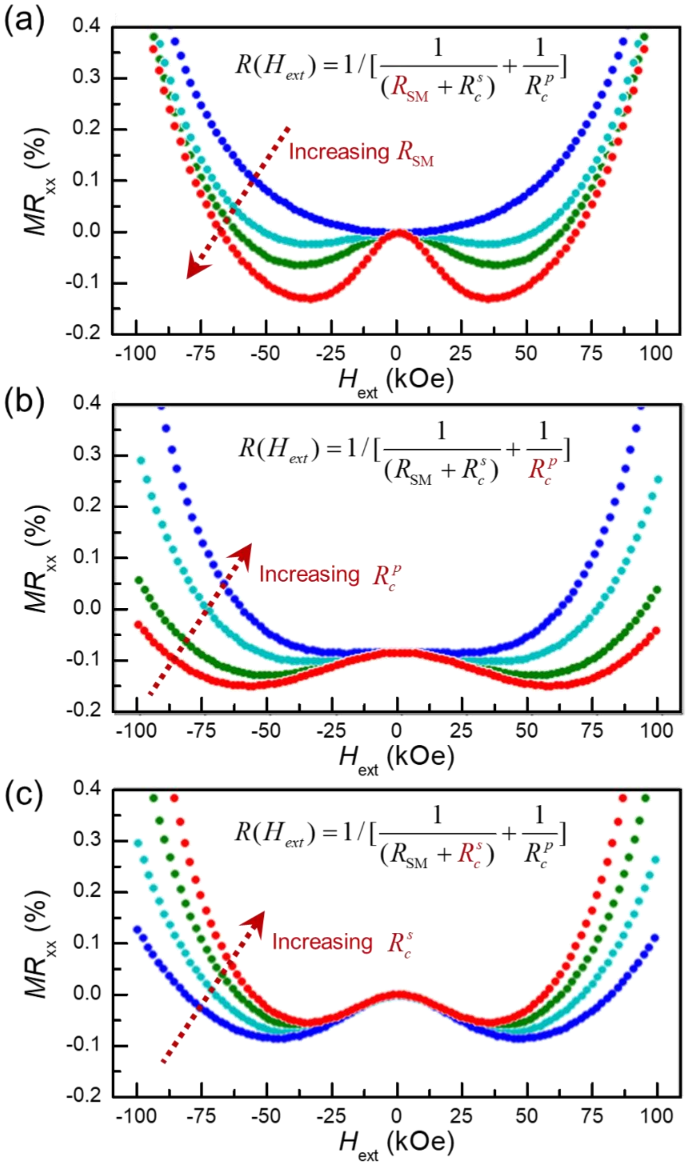

Fig. S7. Three-resistor model to explain NLMR of Pt_3Sn thin films. (a) Change of the total MR with the change of R_{SM}. Negative MR is further enhanced with the increase of R_{SM}. **(b)** and **(c)** MR results for the system with the change of R_c^p and R_c^s, respectively. The change of R_c^s has very small influence on the MR for the low field region and enhance the positive MR for high field region, while the change of R_c^p increase the positive MR for both low and high field region.

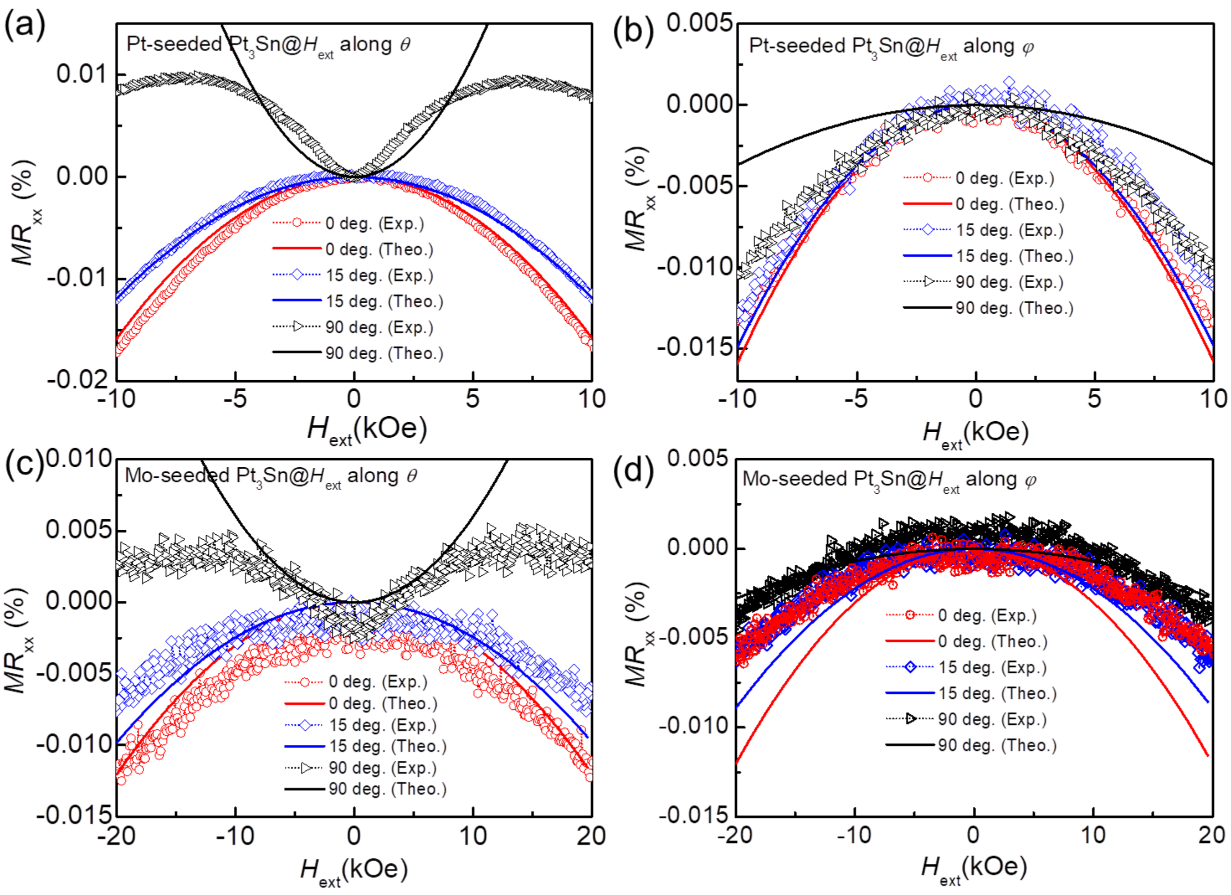

Fig. S8. Experimental and three-resistor model fitting NLMR for Pt-seeded and Mo-seeded Pt_3Sn. (a) and **(b)** Experimentally measured (lines with solid dots) and model fitted (lines with open dots) MR signal for θ and φ dependent measurement of Pt-seeded Pt_3Sn, respectively. **(c)** and **(d)** same as **(a)** and **(b)** for Mo-seeded Pt_3Sn. Weak anti-localization effect is neglected in the three-resistor model fitting.

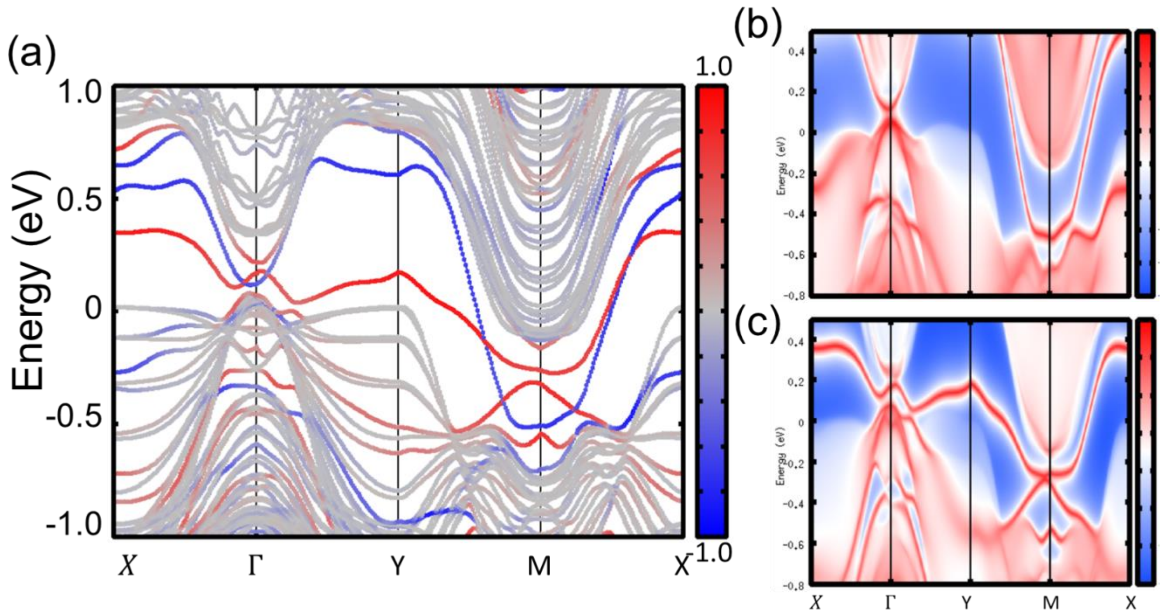

Fig. S9. Topological edge state of pristine Pt_3Sn. (a) Band structure of Pt_3Sn slabs along (001) direction. Periodicity along x and y direction are kept with semi-infinite slab along the z direction. Surface states from two ends are shown in red and blue color with the bulk states shown in grey. Clear surface states can be seen within the bulk gap, confirming the topological insulator feature. **(b)** and **(c)** shows the edge state only band structure for the two surfaces. Red color denotes the contribution from the surface state.

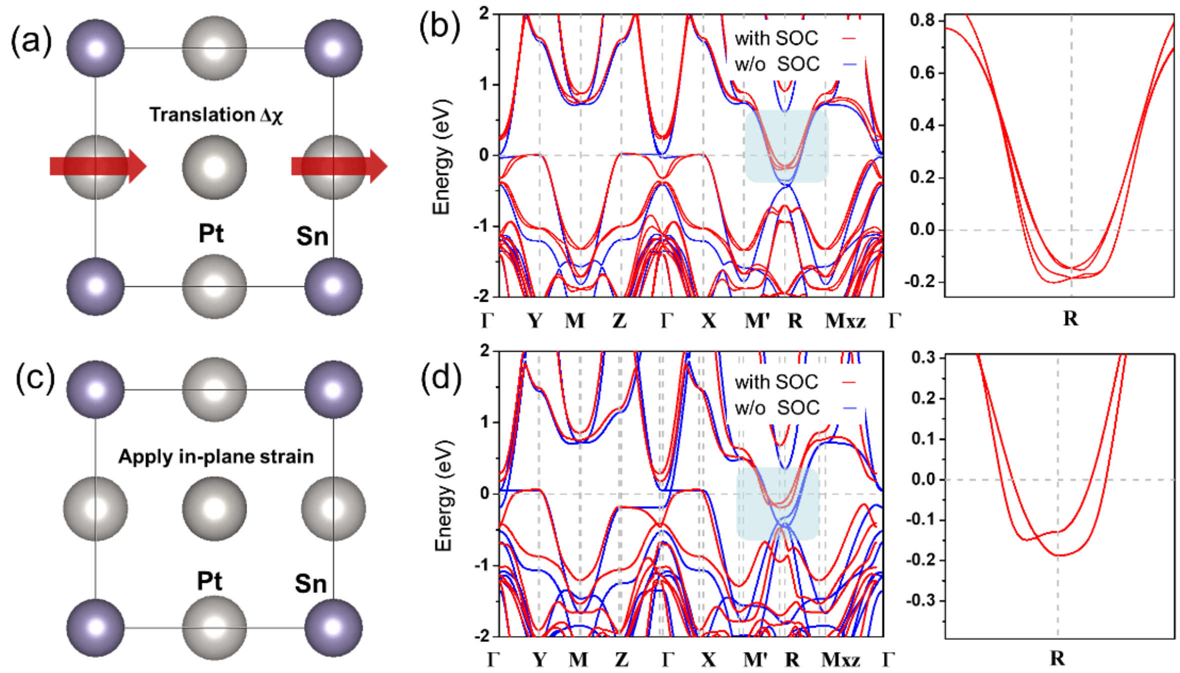

Fig. S10. Robust topological semimetal states of Pt_3Sn against structural perturbation. (a) Crystal structure of Pt_3Sn with Pt atoms shifting toward x direction and **(b)** corresponding band structure with blue shaded area enlarged in right panel to show the formation of Weyl nodes, respectively. Slabs along (001) direction to break the inversion symmetry. **(c)** Crystal structure of Pt_3Sn with in-plane strain and **(d)** corresponding band structure with blue shaded area enlarged in right panel to show the formation of tilted Dirac nodes, respectively.

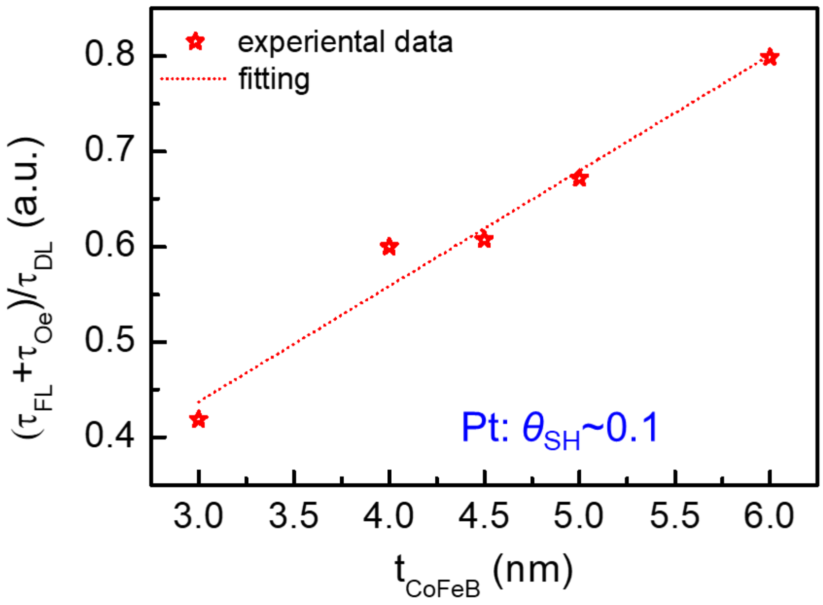

Fig. S11. The spin torque efficiency (θ_{SH}) of the Pt reference thin film characterized by ST-FMR.

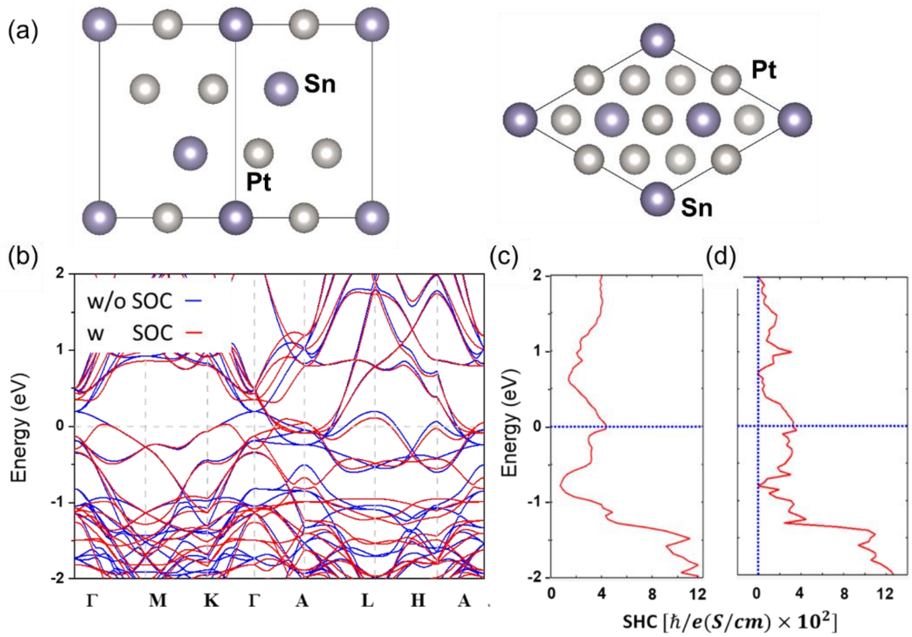

Fig. S12. Spin Hall conductivity of pristine Pt_3Sn with different orientations. (a) Crystal structure of Pt_3Sn superstructure along (111) direction and its **(b)** band structure. Red and blue lines correspond to the band structure with and without SOC, respectively. **(c)** and **(d)** Calculated energy-dependent spin Hall conductivity with ~ 4.34×10^5 $\hbar/2e$ $(\Omega\cdot m)^{-1}$ and ~ 3.6×10^5 $\hbar/2e$ $(\Omega\cdot m)^{-1}$ at Fermi level for pristine Pt_3Sn along (001) and (111) directions, respectively.
